# Correlation between Body Mass Index and Lipid Profile in patients with Type 2 Diabetes attending a tertiary care hospital in Peshawar

**DOI:** 10.12669/pjms.35.3.7

**Published:** 2019

**Authors:** Arshad Hussain, Iftikhar Ali, Waqar Ahmad Kaleem, Fatima Yasmeen

**Affiliations:** 1 *Dr. Arshad Hussain, MRCP. Department of Medicine & Allied, Northwest General Hospital & Research Centre, Peshawar, Khyber Pakhtunkhwa, Pakistan*; 2 *Iftikhar Ali, M.Phil. Department of Pharmacy, University of Swabi, Khyber Pakhtunkhwa, Pakistan., Paraplegic Center, Hayatabad, Peshawar, Khyber Pakhtunkhwa, Pakistan*; 3 *Dr. Waqar Ahmad Kaleem, PhD, Department of Pharmacy, University of Swabi, Khyber Pakhtunkhwa, Pakistan*; 4 *Fatima Yasmeen, M.Sc.(Hon), Nutritionist, Paraplegic Center, Hayatabad, Peshawar, Khyber Pakhtunkhwa, Pakistan*

**Keywords:** Lipid profile, Body mass index, Type 2 diabetes mellitus

## Abstract

**Objective:**

To determine the correlation between body mass index (BMI) and lipid profile in patients with Type 2 Diabetes (T2DM) attending a tertiary care hospital in Peshawar.

**Methods:**

A total of 305 patients (men, 132; women, 173) with T2DM visiting an Outpatient department in Northwest General Hospital and Research Centre, Peshawar from January 2016 to July 2016 were included in this study. The whole blood and sera were analyzed for Glycated hemoglobin (HbA1c), total cholesterol (TC), triglyceride (TGs), high density lipoprotein cholesterol (HDL-C) and low density lipoprotein cholesterol (LDL-C). The correlation of BMI with lipid ratios and individual lipid indices were analysed.

**Results:**

Mean of BMI was 29.29±5.23. Dyslipidemia; increased TC, increased LDL-C, increased triglyceride and decreased HDL-C were noted in 40.7%, 54.1%, 69.5% and 41% respectively. The mean difference of LDL-C (*p*=0.006) was significant between male and female. BMI, TC, TGs, and LDL-C showed no significant correlation where as a significant negative correlation between BMI and HDL-C was observed (r=-0.125, *p*=0.029, R^2^=0.016). The mean values of TC, TG, LDL-C, TC/ HDL-C and LDL/HDL were greater in patients with normal BMI compared to overweight and obese; however, the differences were not significant. HDL-C differed significantly in BMI groups (p=0.040).

**Conclusion:**

A significant negative correlation between BMI and HDL-C was observed, while the correlation between BMI and LDL-C was observed to be insignificant. HDL-C was found significantly higher in patients with normal BMI. These results are important to indicate that there is modest impact of BMI on lipid profile. Therefore, assessment and management for altered blood lipids should not be based on a patient’s body weight or BMI.

## INTRODUCTION

Diabetes is globally a fast growing public health concern, with an enormous effect on not only individuals and health care system, but also the economy of nations.^1^ In line with the latest information from the International Diabetes Federation, 451 million adults worldwide have diabetes; this number is predicted to touch the 693 million figure by 2045.^2^

Globally, Asian countries have the highest number of people with diabetes. Because of genetic variation and high vulnerability to environmental factors, classified via a low BMI, upper body high adiposity, excessive body fat proportion and a high degree of insulin resistance, the population of Asian Subcontinent faces higher risk for diabetes and its complications.^3^,^4^ BMI is frequently used to categorize individuals as underweight, normal, overweight and obese.^5^ It has been extensively described that BMI is a strong predictor of heart diseases and T2DM. Association of lipid profile is reported with lifestyle, intra-abdominal adiposity, obesity and BMI.^6^

Studies have shown a direct relationship between increasing BMI and raised TC, LDL-C, and TG and an inverse correlation with HDL-C. This correlation between BMI and lipoprotein levels, especially LDL-C, has been proposed to be a strong contributing risk factor for cardiovascular diseases in obese individual. Nevertheless, the sample size of obese and morbidly obese individuals in these studies is lacking to draw a conclusion regarding the expected lipid parameters in this population.^7^,^8^ Recently conducted observational studies validated a correlation between BMI and TG or HDL-C in obese patients, except LDL-C levels. These results have raised the question of a possible “obesity paradox” where LDL-C levels may elevate or decline with extreme BMI levels.^9^,^10^ Therefore, we sought to evaluate the correlation between body mass index and lipid profile in people with T2DM attending a tertiary care hospital in Peshawar, Pakistan.

## METHODS

This cross sectional study was carried out from January 2016 to July 2016. Patients (T2DM) visiting an Outpatient department in Northwest General Hospital and Research Centre, Hayatabad, were included in the study. The hospital is a tertiary care facility, located in Peshawar. It serves patients from the whole province as well as across the border from Afghanistan. The study protocol was approved by the ethics committee of Northwest General Hospital and Research Center [Ref No. NwGH/Res/approv/9].

Blood samples (Venous) were obtained and the serum was used for analyzing HbA1c, Total cholesterol (TC), HDL-C; TG and LDL-C, using an auto analyzer (“Roche/Hitachi 912/Modular Analyzers: CAN 435”.). HbA1c was measured by “ARCHITECT c 4000 analyzer (Abbott Diagnostics)”. The National Cholesterol Education Program Adult Treatment Panel III (NCEP ATP III) guidelines were referred for the serum lipid reference level.^11^ As per the guidelines, “hypercholesterolemia is defined as TC >200 mg/dl, high LDL-C when the value is >100 mg/dl, hypertriglyceridemia when TG is >150 mg/dl and low HDL-C as a value <40 mg/dl”. “Dyslipidemia was defined as the presence of one or more abnormal serum lipid concentrations”.

Patient’s weights were taken to the nearest 0.1kg. The scale was positioned on a hard surface. The patients were then asked to wear light garment and stand in the center of the platform bare footed with their weight distributed evenly to both feet. Patients heights were measured using wall mounted stadiometer to the nearest 0.5cm. Patients were requested to stand upright with their back to the height rule. BMI was calculated as weight kg/height squared (kg/m²) and all patients according to their BMI were divided into three groups. Patients with BMI≥25kg/m² were considered obese.

### Statistical analysis

SPSS Version 21.0 was used for data analysis. Pearson’s correlation was used to see various correlations. Mean comparison of different parameters was done through independent t-test and ANOVA. P-value less than 0.05 were considered statistically significant whereas value of less than 0.01 was taken highly significant. HbA1c value was measured in percentage of total hemoglobin, all other parameters values were expressed in mg/dl. All values are presented as mean ± SD or standard error of mean.

## RESULTS

Of the 305 patients, 132 (43.3%) were males and 173 (56.7%) were females. Mean age (SD) was 50.19±10.16 years. The ages of the patients ranged from 17-82 years. Table-I shows that the mean (SD) of HbA1c, TC, TGs, LDL-C and HDL-C were 09.72 ± 2.62, 189.32 ± 46.68, 218.90 ± 126.24, 107.95 ± 47.10 and 52.32 ± 33.11, respectively. The age, blood lipoproteins and triglycerides, HbA1c and the BMI were stratified by gender. LDL-C differed significantly while the rest of the parameters did not show significant difference in a gender wise manner.

**Table-I:** Comparison of age, BMI, HbA1c and blood lipoproteins and triglycerides among male and female patients.

Parameters	Male (N=132)			Female (N=173)			p-value

	Mean	SD	Range	Mean	SD	Range
Age (years)	50.83	11.03	22-82	49.70	10.27	17-77	0.359
BMI(Kg/m^2^)	29.24	04.70	18-43	29.32	5.57	17-42	0.889
HbA1c (%)	09.43	02.70	5.5-17	9.91	2.53	5.5-17.1	0.112
TC(mg/dl)	184.76	42.26	94-306	192.80	49.63	96-318	0.136
TG (mg/dl)	224.05	139.53	48-1046	214.97	115.33	65-779	0.535
LDL-C (mg/dl)	99.42	47.65	20-265	114.46	45.75	43-240	0.006
HDL-C (mg/dl)	54.68	37.55	13-179	50.51	29.26	18-207	0.277
TC/HDL-C	4.43	2.09	1.33-15.38	4.37	1.36	1.32-8.35	0.744
LDL-C/HDL-C	2.59	1.51	0.18-8	2.69	1.23	0.17-5.82	0.496

The numbers and percentages of abnormal BMI, HbA1c, TC, TG, LDL-C and HDL-C were calculated to be 89.5%, 82.6%, 40.7%, 69.5%, 54.1% and 41%, respectively as shown in Table-II. Normal BMI and HbA1c were defined by World Health Organization (WHO) recommended^5^ Asia and Asia-pacific region cutoffs and American diabetes association (ADA)^12^ references correspondingly. NCEP ATP III, criteria was adapted for normal lipid ranges.^11^

**Table-II:** Percentage of normal and abnormal BMI, HbA1c and lipid values in study sample.

Variables	Normal	Abnormal
BMI(Kg/m^2^)	32(10.5%)	273(89.5%)
HbA1c (%)	53(17.4%)	252(82.6%)
TC(mg/dl)	181(59.3%)	124(40.7%)
TG (mg/dl)	93(30.5%)	212(69.5%)
LDL-C (mg/dl)	140(45.9%)	165(54.1%)
HDL-C (mg/dl)	180(59%)	125(41%)

**Fig.1:**
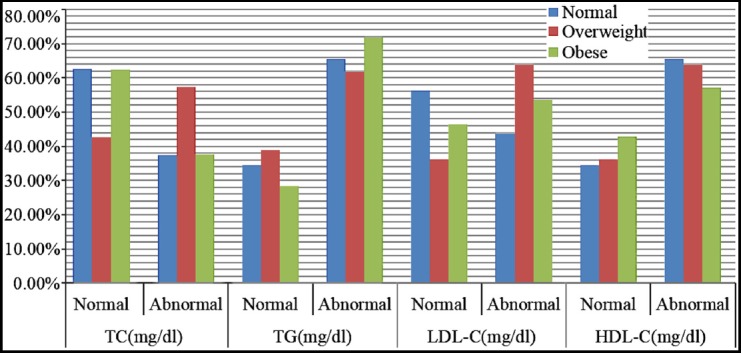
Percentage of normal and abnormal lipid parameters to three BMI groups.

The percentages of normal and abnormal lipid profile in the BMI groups can be seen in Fig.1. The highest number of normal lipid parameters was observed with normal BMI, with 62.5%, 34.4%, 56.3% and 34.4% of the patients having normal values for TC, TG, LDL-C and HDL-C respectively. Similarly in obese BMI, with 62.4%, 28.3%, 46.5% and 42.9% patients having normal values for TC, TG, LDL-C and HDL-C respectively.

The Pearson’s co-efficient between the BMI and the lipid parameters obtained only one significant correlation as shown in Table-III. The BMI showed significantly negative correlation (r =-0.125, *p*=0.029, R^2^=0.016) with HDL-C value, while the others parameters like TC(r=-0.052, *p*=0.367, R^2^=0.003), TG(r=-0.006, *p*=0.919, R^2^=0.000), LDL-C(r=-0.021, *p*=0.721, R2=0.000), LDL-C/HDL-C (r=0.032,*p*=0.576, R^2^=0.001) and TC/HDL-C (r=0.097,*p*=0.091, R^2^=0.009) did not show any significant correlation with BMI.

**Table-III:** Correlation analysis between BMI and lipid parameters.

		BMI	TC	TG	HDL-C	LDL-C
TC	r	-0.052				
	p	0.367				
TG	r	-0.006	0.343**			
	p	0.919	0.000			
HDL-C	r	-0.125*	0.235**	-0.064		
	p	0.029	0.000	0.268		
LDL-C	r	-0.021	0.647**	0.072	-0.420**	
	p	0.721	0.000	0.210	0.000	
LDL-C/HDL-C	r	0.032	0.381**	0.197**	-0.681**	0.830**
	p	0.576	0.000	0.001	0.000	0.000
TC/HDL-C	r	0.097	0.352**	0.358**	-0.684**	0.592**
	p	0.091	0.000	0.000	0.000	0.000

* “Correlation is significant at the 0.05 level (2-tailed)”,

** “Correlation is significant at the 0.01 level (2-tailed)”.

Patients were divided into three groups based on their body mass index (BMI); group one consisted of patients with normal BMI (≤22.9kg/m^2^), the group two consisted of patients with BMI value (23.0–24.9kg/m^2^) and group three (≥25kg/m^2^). Group one was noted to have higher values of TC (201.79±47.94), TG, (234.94±167.03) LDL-C (120.98±50.56), TC/HDL-C (4.61±1.87) and LDL-C/HDL-C (2.90±1.46), compared to group two and three respectively as shown in Table-IV, however the differences were not statistically significant. Moreover the mean value of HDL (66.16±45.89) was higher in patients with normal BMI; this difference across the BMI groups was statistically significant (p=0.040).

**Table-IV:** Comparison of Age, HbA1c and lipid parameters with BMI groups.

Parameters	Normal (N=32)				Overweight (N=47)				Obese (N=226)				p-value

	Mean	SD	Range	SE	Mean	SD	Range	SE	mean	SD	Range	SE	
Age (years)	47.28	7.76	22-65	1.37	48.49	11.82	17-82	1.72	50.95	10.61	22-77	0.706	0.091
HbA1c (%)	10.54	3.42	5.7-17.0	0.61	09.47	2.55	5.5-15.8	0.37	9.63	2.49	5.5-17.1	0.165	0.147
TC (mg/dl)	186.91	51.94	102-312	9.81	201.79	47.94	94-318	6.99	187.07	45.43	96-318	3.02	0.138
TG (mg/dl)	203.56	113.74	62-538	20.11	234.94	167.03	58-779	24.36	217.73	118.13	48-1046	7.86	0.537
LDL-C (mg/dl)	99.13	53.16	33-218	9.40	120.98	50.56	30-265	7.37	106.50	45.130	20-229	3.002	0.084
HDL-C (mg/dl)	66.16	45.89	26-179	8.11	52.36	28.58	20-165	4.17	50.35	31.51	13-207	2.096	0.040
TC/HDL-C	3.72	1.85	1.40-7.26	0.33	4.61	1.87	1.33-8.35	0.27	4.45	1.78	1.32-15-38	0.118	0.070
LDL-C/HDL-C	2.28	1.62	0.25-4.81	0.286	2.90	1.46	0.18-5.19	0.214	2.64	1.29	0.17-8.00	0.086	0.139

SE; standard error. Group-1: Normal BMI; Group-2: Overweight BMI; Group-3: Obese BMI.

## DISCUSSION

Research literature support a relationship between BMI and TG, and the relationship of blood lipids and body fat distribution has been under discussion over the past few decades.^3^,^13^ Body fat and blood lipids have been observed to be key determinants of metabolic disorders, like cardiovascular diseases (CVD), diabetes, dyslipidemia, hypertension, hyperinsulinemia and elevated serum uric acid.^14^,^15^

Dyslipidemia, a well known risk factor for cardiovascular manifestations, is mostly observed in the population of the Asian continent. People with T2DM have an increased cardiovascular morbidity and mortality, and are affected more by CVD compared with non diabetics. Prompt recognition and management of DM associated dyslipidemia might be one step in controlling the risk of CVD.^3^,^15^,^16^

Obesity, which is considered to be potentially linked with abnormal lipids and poor cardiovascular outcomes, is becoming a highly prevalent condition in Pakistan.^17^ The current study intends to evaluate the correlation between BMI and lipids in patients with T2DM in a Khyber Pakhtunkhwa population; an area from where information in this regard is up till now not available.

In this study, 305 diabetic patients attending the outpatient department (diabetic clinic) at the Northwest General Hospital and Research Centre, Peshawar were randomly selected for the study. The participants were already diagnosed as having T2DM and were under treatment at the diabetic clinic. The patients included 132 (43.3%) men and 173 (57.7%) women. The female population was more than that of the male counterpart. This compares well with a study on WHO global data which stated that the prevalence ratio of DM between men and women varies markedly, with no consistent trend.^18^

Age, BMI, HbA1c and lipids values were stratified in gender wise manner; in male patients mean values of BMI and TC were slightly lower and that of TG and HDL-C were higher compared to female patients but the mean differences were not statistically significant which is consistent with previous studies results in TG^15^ and TC.^6^ However the mean difference of LDL-C in male and female patients was statistically significant. This is in agreement to the findings of a study which showed similar result in LDL-C values between men and women, although the mean values of TG and HDL-C were differ significantly whereas TC was comparable in both genders.^19^ In a study by Omotoye FE et al. which showed that mean TC, TG, and LDL-C were elevated more among the T2DM female patients than males.^6^

Following the WHO, ADA and NCEP ATP III criteria for BMI, HbA1c and Lipid profile values. The most common lipid abnormality was seen in TGs with 69% of the study participants, followed by the LDL-C(54.1%). This result is in agreement with published studies in Northwest Ethiopia (63.5%), Hyderabad-India (60%) and Sudan (48.8%).^20^ These findings may be due to the increased secretion of LDL-C by the liver and slow removal of TGs rich lipoproteins, as well as raised levels of substrates for TG production from augmented mobilization of free fatty acid (FFA) from adipose tissue in people with diabetes.^21^ High TG levels are a prominent lipid abnormality in T2DM and also occur in individuals with pre-diabetes states. A fasting TG level of >150 mg/dl is one of the benchmark for characterizing peoples at high risk for CVD and T2DM. Our results showed raised LDL-C and low HDL-C levels in DM patients. These results are in agreement to Asian Pacific Cohort Studies Collaboration.^22^ These findings are thought to be due to differences in genetic makeup, differences in life style and the management of specific population of DM being studied.

This study showed that in the study population mean BMI was not different in males and females (29.24 vs. 29.32) kg/m^2^. These BMI results were higher than the previously published mean BMI of participants from an urban community in Yemen (23.9 ± 5.1) kg/m^2^ and 21.8 ±8.9) kg/m^2^ in females and males, respectively^23^ and also the work of Al-Sharafi which showed that the overall mean BMI was considerably higher in females than in males (28 vs. 25.4).^24^

In our study two patients were underweight (17.5 kg/m^2^) which was considered normal. Whereas, 10.5% were normal weight, 15.4% were overweight and obese (BMI>25) accounted for 74.1% of the total investigated population with diabetes mellitus. This figure was lower than that of a study by Bansal P et al.^25^ but higher than the findings of a previous study in Yemen that overweight and obesity accounted only for 26.2% of patients with T2DM aged 20-65.^24^ The international data Analysis with reference to the relationship between BMI and both morbidity and mortality recommended that the association of BMI with most diseases was rather continuous^26^ and commonly, women had a higher mean BMI than men.^27^ With regards to relationship between BMI and lipid profile this study showed that BMI had a negative correlation (r =-0.125, *p*=0.029) with HDL-C value while the others parameters like TC(r=-0.052, *p*=0.367), TG(r=-0.006, *p*=0.919), LDL-C(r=-0.021, *p*=0.721) did not reveal any correlation with BMI.Our results are different from other similar studies. Results of a study conducted in Korea described that there was a positive correlation between BMI with TC and LDL-C respectively; whereas a study from India illustrated the existence of only BMI vs. LDL-C correlation.^28^,^29^ A weak negative correlation of HDL-C with BMI was also reported by a similar study conducted in Nigeria.^6^ Likewise in a study by Shamai et al there was an association between BMI, HDL-C and TG.^7^

In this study T2DM patient with normal BMI when compared to overweight and obese BMI did not show significant differences in the mean values of TC, TGs and LDL-C except HDLC. In a study by Yadav NK et al., reported that obese T2DM patients, in comparison with on-diabetic obese control patients revealed statistically significant increase in the levels of TC, TGs, LDL-C whereas HDL-C levels in the two groups did not show statistically significant difference.^30^ Another study on Iraqi diabetic premenopausal women showed that high BMI is consistently coupled with abnormal lipid profile marked by elevated TGs and LDL-C, and low HDL-C.^13^ Comparison of HDL-C levels showed that Group one individuals had the most favourable values (66.16 ± 45.90). This was followed by individuals in Group two (52.36 ± 28.58) and Group three (50.35 ± 31.51). This difference in HDL-C levels across the three groups was significant (p < 0.040). Our results are consistent with a study by Bora K et al.^31^

Both body fat and lipid parameters have been revealed to be the significant predictors for metabolic disturbances including diabetes, dyslipidaemia, hypertension, hyperinsulinaemia, and cardiovascular diseases. Lipid profiles association is reported with lifestyle, age, intra-abdominal adiposity, Obesity and BMI.^6^

### Limitations of the study

The contribution of diet and socio-economic factors in influencing lipid profile and obesity were not considered. In addition, a randomly drawn larger sample would have been more advantageous. Constraint resources and lack of time was the chief causes for these limitations. Similarly waist circumference (WC) was not measured.

## CONCLUSION

This study showed high percentage of abnormal TG. LDL-C level was observed to be significantly higher in female. A significant negative relationship between HDL-C and BMI level was also seen, while the correlation between LDL-C and BMI was observed to be insignificant. HDL-C was found significantly higher in patients with normal BMI. These results are imperative as they back up that there is modest impact of BMI on lipid profile. Diabetic patients are more likely to have dyslipidemia which is a key determinant for atherosclerosis and CVD. Normal BMI significantly improves dyslipidemia in T2DM patients. Further studies with large sample size are needed to identify the causes of obesity that would help in better understanding of its influence on lipid profile.

### Authors’ Contributions

**AH and IA:** Conception & design.

**AH:** Data collection.

**AI:** Data analysis and interpretation/results.

**AI, WAK and FY:** Manuscript drafting and writing.

**AH, IA and FY:** Language editing/appropriateness, critical revision.

All authors have read and approved the final version of the paper.
